# Targeting CD133 reverses drug-resistance via the AKT/NF-κB/MDR1 pathway in colorectal cancer

**DOI:** 10.1038/s41416-020-0783-0

**Published:** 2020-03-16

**Authors:** Zeting Yuan, Xin Liang, Yueping Zhan, Ziyuan Wang, Jian Xu, Yanyan Qiu, Jie Wang, Yijun Cao, Van-Minh Le, Hai-Trieu Ly, Jianhua Xu, Wei Li, Peihao Yin, Ke Xu

**Affiliations:** 1 Interventional Cancer Institute of Chinese Integrative Medicine, Putuo Hospital, Shanghai University of Traditional Chinese Medicine, 200062 Shanghai, China; 2 Shanghai Putuo Central School of Clinical Medicine, Anhui Medical University, 230032 Hefei, China; 3 State Key Laboratory of Bioreactor Engineering & Shanghai Key Laboratory of New Drug Design, School of Pharmacy, East China University of Science and Technology, 130 Meilong Rd, 200237 Shanghai, People’s Republic of China; 4 Central Laboratory, Putuo Hospital, Shanghai University of Traditional Chinese Medicine, 200062 Shanghai, China; 5 Department of Pathology, Shuguang Hospital, Shanghai University of Traditional Chinese Medicine, Shanghai, China; 6 Department of General Surgery, Putuo Hospital, Shanghai University of Traditional Chinese Medicine, 200062 Shanghai, China; 7 Research Center of Ginseng and Medicinal Materials (CGMM), National Institute of Medicinal Materials, Ho Chi Minh City, 70000 Vietnam

**Keywords:** Cancer stem cells, Molecular biology

## Abstract

**Background:**

Recent studies have shown that multidrug resistance may be induced by the high stemness of cancer cells. Following prolonged chemotherapy, MDR protein 1 (MDR1) and CD133 increase in CRC, but the relationship between them is unclear.

**Methods:**

The relationship between MDR and CSC properties in CRC was determined via CCK-8 assay, apoptosis assay, DOX uptake and retention, immunohistochemistry, immunofluorescence and flow cytometry. The correlations between their expression levels were evaluated using Spearman’s rank statistical test and the Mann-Whitney test. Furthermore, the effect of CD133 on the repression of the AKT/NF-κB/MDR1 signalling pathway was investigated in vitro and in vivo.

**Results:**

We found that CD133 increased with the emergence of drug-resistance phenotypes, and the high expression of MDR1/P-gp was consistently accompanied by positive expression of CD133 as demonstrated by the analysis of patient samples. Up- or downregulation of CD133 could regulate MDR via AKT/NF-κB/MDR1 signalling in CRC. A rescue experiment showed that the AKT/NF-κB signalling pathway is the main mechanism by which CD133 regulates MDR1/P-gp expression in CRC.

**Conclusions:**

Taken together, our results suggest that targeting CD133 reverses drug resistance via the AKT/NF-κB/MDR1 pathway and that this pathway might serve as a potential therapeutic target to reverse MDR in CRC.

## Background

Multidrug resistance (MDR) is the main cause of chemotherapy failure and disease progression in colorectal carcinoma (CRC) patients.^1,2^ The mechanisms of MDR are varied and include increased drug efflux, reduced drug uptake, effects on membrane lipids, increased drug metabolism, changes in drug targets, inhibition of programmed cell death (apoptosis), induction of DNA damage repair and alterations of the cell cycle and associated checkpoints.^3^ Overexpression of ABC transporters, such as MDR1/P-gp, which limits the long-term effective use of chemotherapeutic drugs, is the main cause of MDR.^4^

Cancer stem cells (CSCs) play important roles in tumour survival, proliferation, metastasis and recurrence.^5,6^ Overall, CSCs maintain the viability of tumour cells through self-renewal and infinite proliferation. These cells are also believed to be associated with chemotherapeutic resistance.^7^ CRC has several key stem cell markers, including CD133, CD44, CD26, CD166 and ALDH1.^8,9^ Experimental evidence has shown that the expression of CSC markers is positively correlated with drug resistance.^10^ Conversely, decreased expression of CSC markers resulted in decreased expression of ABC efflux pumps and increased tumour sensitivity to chemotherapy.^11^ Overexpression of ABC transporters plays an important role in chemoresistance in colorectal CSCs.^12,13^ Inhibition of CD133 enhanced Cis-KATO-III cell sensitivity to cisplatin through regulation of the PI3K/AKT/mTOR signalling pathway in gastric cancer cells.^14^

Akt serine-threonine kinase 1 (AKT) acts as a target and effector of downstream phosphatidylinositol 3-kinase (PI3K).^15^ The PI3K/AKT pathway is related to MDR1/P-gp expression.^16,17^ PI3K/AKT activates NF-κB, which induces MDR1/P-gp expression by binding to its promoter.^18^ The PI3K/AKT signalling pathway is activated by the CD133/p85 interaction and promotes tumorigenesis of glioma stem cells.^19^ CD133 activates the PI3K/AKT signal transduction pathway by directly interacting with PI3K-p85 in gastric cancer cells.^20^ CD133-positive colon CSCs often exhibit excessive activation of the PI3K/AKT pathway.^21^ However, there is no research on the molecular mechanism of the PI3K/AKT signalling pathway between MDR and CD133 + CRC cell properties.

In this study, we investigated the relationship between MDR and CSC properties in CRC. We found that CD133 increased with the emergence of drug-resistance phenotypes, and targeting CD133 reversed drug resistance via the AKT/NF-κB/MDR1 pathway, further providing a potential therapeutic target to reverse MDR in CRC.

## Methods

### Cell lines and reagents

The human colon cancer cell lines LoVo and HCT8 were obtained from the Cell Bank of the Chinese Academy of Sciences. DOX-resistant LoVo/ADR and HCT8/ADR cell lines were purchased from Shanghai Yan Sheng Industrial Co., LTD. All cell lines were used in the reversal study and were cultured in RPMI-1640 or F12K containing 10% FBS at 37 °C in a humidified atmosphere of 5% CO_2_, as described previously.^22^ All DOX-resistant cells were seeded in medium containing 1 μM DOX to maintain the drug-resistance phenotype. DOX, MMC, VCR and CTX were obtained from Sigma–Aldrich Chemical Co. (St. Louis, MO, USA). The CD133-knockdown shRNA plasmid was purchased from Santa Cruz Biotechnology (Santa Cruz, CA), and the AKT overexpression plasmid, the NF-κB/p65 overexpression plasmid and the MDR1 promoter plasmid were purchased from Addgene (Cambridge, MA).

### Tissue samples

Human CRC samples and corresponding non-tumourous colon (NC) samples were collected at the time of surgical resection at Putuo Hospital, Shanghai University of Traditional Chinese Medicine, P.R. China, from January 2010 to December 2011, as described previously.^23^ Written informed consent was obtained from the patients before sample collection in accordance with institutional guidelines, and the study was approved by the Committees for the Ethical Review of Research at the Putuo Hospital, Shanghai University of Traditional Chinese Medicine, P.R. China. All procedures were performed in accordance with the approved guidelines. All patients had a histological diagnosis of CRC and underwent radical resection. None of the patients included in the study had received neoadjuvant therapy before surgery. The samples were immediately snap frozen in liquid nitrogen and stored at −80 °C.

### Cell viability and apoptosis assays

Colon cancer cells were plated in 96-well plates and treated with various chemotherapeutic agents for the indicated times. After 48 h, cell viability was assessed using a CCK-8 assay (Dojindo Molecular Technologies, Inc., MD, USA) as described previously.^23^ The absorbance of each well at 490 nm was read on a spectrophotometer (Bio-Rad, Hercules, CA.). Cell viability was calculated as a ratio of the OD values of drug-treated samples to those of controls. For apoptosis, an annexin V-FITC apoptosis detection kit (Invitrogen, NY, USA) was used according to the manufacturer’s instructions.

### DOX uptake and retention

As described previously,^24^ to visualise the uptake of DOX, 1 × 10^4^ colon cancer cells were seeded in 8-well chamber slides (BD, NJ, USA) and cultured for 24 h. The cells were washed twice with serum-free medium and incubated with DOX (1 μg/ml) in serum-free medium for 1 h at 37 °C. The cells were then washed with phosphate-buffered saline (PBS) and incubated with culture medium. At 6 h, the supernatant was removed, and the cells were fixed in 4% paraformaldehyde (4 °C, 10 min). Nuclei were stained with DAPI (Invitrogen, NY, USA) (20 °C, 5 min). The cells were visualised with a Zeiss 510 confocal laser-scanning microscope using a 63 N (NA1.32) objective.

To investigate the cellular DOX retention level, 5 × 10^3^ cells/well were seeded into 48-well plates and incubated overnight. Culture medium (negative control) and DOX (1 μg/ml) were added to each well. After 1 h, the cells were washed with PBS and incubated with culture medium. At 6 h, the supernatant was removed, and the cells were washed with ice-cold PBS without iron and lysed with PBS containing 1% Triton X-100. DOX concentrations in the cell lysates were measured using a Wallac VICTOR2 1420 multilabel counter (Perkin Elmer, MA, USA) at an excitation wavelength of 478 nm and an emission wavelength of 594 nm. Cellular DOX uptake is expressed as nanomoles per milligram of protein. The protein concentrations of the cell lysates were determined with a BCA Protein Assay Kit (Thermo Fisher Scientific Co., MA, USA).

### Immunofluorescence

Cells cultured on glass cover slides were fixed in 4% paraformaldehyde/PBS for 10 min, permeabilised in 0.2% Triton X-100 for 20 min, and blocked in 100% FBS for 1 h. The fixed cells were incubated with a primary antibody specific for CD133, NF-κB/p65 or P-gp (Cell Signaling Technology, Inc.) and a secondary antibody. Images were obtained by using a confocal laser-scanning microscope (LSM 510; Carl Zeiss, Inc.).

### Quantitative RT-PCR

To prepare total RNA from tissues, 5-mm^3^ sections of each sample were cut, and the frozen tissues were finely ground. The tissue pieces were then subjected to RNA extraction with TRIzol (Invitrogen). Total RNA was also extracted from cultured colon cancer cells with TRIzol (Invitrogen). The concentration of total RNA was quantitated by measuring the absorbance at 260 nm. For SYBR Green-based quantitative PCR amplification, the reactions were performed in a 20 ml reaction volume containing SYBR Green PCR Master Mix (Applied Biosystems). The relative expression levels of each cell line in each group were measured using the 2^−ΔΔCt^ method, as described previously. The relative expression levels of tissues were measured using the 2^-ΔCt^ method. The primer sequences were as follows: MDR1, 5’-AACGGAAGCCAGAACATTCC-3’ and 5’-AGGCTTCCTGTGGCAAAGAG-3’; CD133, 5’-TTCTATGCTGTGTCCTGGGGC-3’ and 5’-TTGTTGGTGCAAGCTCTTCAAGGT-3’; and GAPDH, 5’-CTCCATCCTGGCCTCGCTGT-3’ and 5’-GCTGTCACCTTCACCGTTCC-3’.

### Luciferase activity assay

Cells (2 × 10^4^) were co-transfected with 500 ng of plasmid containing the MDR1 promoter (pGL2-MDR1-promoter-luc) with CD133 overexpression or knockdown. Each sample was co-transfected with 50 ng of the pRL-SV40 plasmid expressing Renilla luciferase to monitor the transfection efficiency. Luciferase activity was measured 48 h after transfection with the Dual-Luciferase Reporter Assay System (Promega, Wisconsin, USA). The relative firefly luciferase activity was normalised to the Renilla luciferase activity.

### Western blot analysis

Proteins were resolved via SDS/PAGE and subjected to immunoblot analysis with specific antibodies (Cell Signaling Technology, US) as described previously.^25^ All antibodies were used at a 1 mg/ml working concentration in PBS with 5% dried milk. The membranes were further probed with horseradish peroxidase (HRP)-conjugated rabbit anti-mouse IgG (Santa Cruz, 1:2000), and the protein bands were visualised using enhanced chemiluminescence (Amersham Pharmacia Corp., Piscataway, NJ). Quantification of protein bands was performed using ImageJ software.

### In vivo xenograft model

Twenty male athymic nude mice (SPF, a weight of 18–22 g nude mouse) (Songlian Experimental Animal Institute, Songjiang District, Shanghai) were acclimated and housed in a clean grade room at 21 ± 1^o^C and 60 ± 5% humidity, under a 12 h light/12 h dark cycle with free access to water and food in a specific pathogen-free environment. Animal husbandry protocols were followed where animals were monitored daily according to humane endpoint guidelines by experimental staff and independently by animal husbandry staff, including in house veterinarians. All animals were assessed to be healthy and free of disease prior to tumour implantation. Colon cancer cells (1 × 10^6^) were injected into the flanks of male athymic nude mice (4–5 weeks old). Two weeks after injection, DOX (0.5 mg/kg) was administered by intraperitoneal (i.p.) injection every 5 days per week for 4 weeks. Tumour volumes were measured at the beginning of the treatment and every 4 days until the mice were euthanised. Animals were humanely euthanised on day 28, and tumour tissues were harvested, weighed, and then immediately fixed in formalin for immunohistochemistry. The estimated tumour volumes (V) were calculated by the formula V = W^2^ × L × 0.5, where W represents the largest tumour diameter in centimetres and L represents the next largest tumour diameter. For animal studies, four treatment groups (1. LoVo/ADR^ctrl^ + normal saline, 2. LoVo/ADR^CD133 KD^ + normal saline, 3. LoVo/ADR^ctrl^ + DOX, 4. LoVo/ADR^CD133 KD^ + DOX) were used, each with five animals. Each experimental group was confined to a separate cage. Cages were selected for a particular treatment at random at the start of treatment (all treatments were started at the same time). All groups were assessed at the same time. Tumours were allowed to establish until they were palpable prior to treatments. Animals were euthanised by carbon dioxide asphyxiation followed by cervical dislocation to ensure death. All protocols were approved and supervised by the Institutional Animal Care and Use Committee of Putuo Hospital, Shanghai University of Traditional Chinese Medicine, P.R. China. All animal studies were conducted in accordance with the National Institutes of Health Guidelines for the Care and Use of Laboratory Animals.

### Immunohistochemistry

As described previously,^23^ Tissues were fixed in 10% formalin, embedded in paraffin, and sectioned (5-mm thickness). TUNEL, CD133 and P-gp immunohistochemistry was conducted as follows: slides were deparaffinised and incubated for 10 min with 3% H_2_O_2_ in water to quench endogenous peroxidase activity. The heat-induced antigen retrieval method was used for the detection of antigens. Tissues were incubated with 5% normal rabbit serum in TBS (0.05 M Tris-HCl, 0.5 M NaCl, pH = 7.4) for 30 min at room temperature and incubated with primary antibodies in TBS for 60 min at 37 °C. The indirect avidin–biotin–peroxidase method was applied using the appropriate secondary antibodies for 30 min at room temperature. The EnVision (K4007, Dako) signal enhancement system was used to develop the bound antibodies. Sections were counterstained with Harris haematoxylin, dehydrated and mounted. For quantification, 30 random images (×400) per experimental group were captured with a microscope (Leica, Wetzlar, Germany).

### Statistical analysis

Each experimental value is expressed as the mean ± standard deviation (SD). Statistical analysis was performed using a *t*-test to evaluate the significance of the differences between cell groups, with significance accepted at *p < 0.05 and **p < 0.01. All data points represent the mean value of triplicate measurements. Statistical analysis of tissue samples was performed using Spearman’s rank correlation and the Mann-Whitney test to evaluate the significance of differences between groups.

## Results

### CD133 overexpression in ADR-resistant CRC cells

**Fig. 1:**
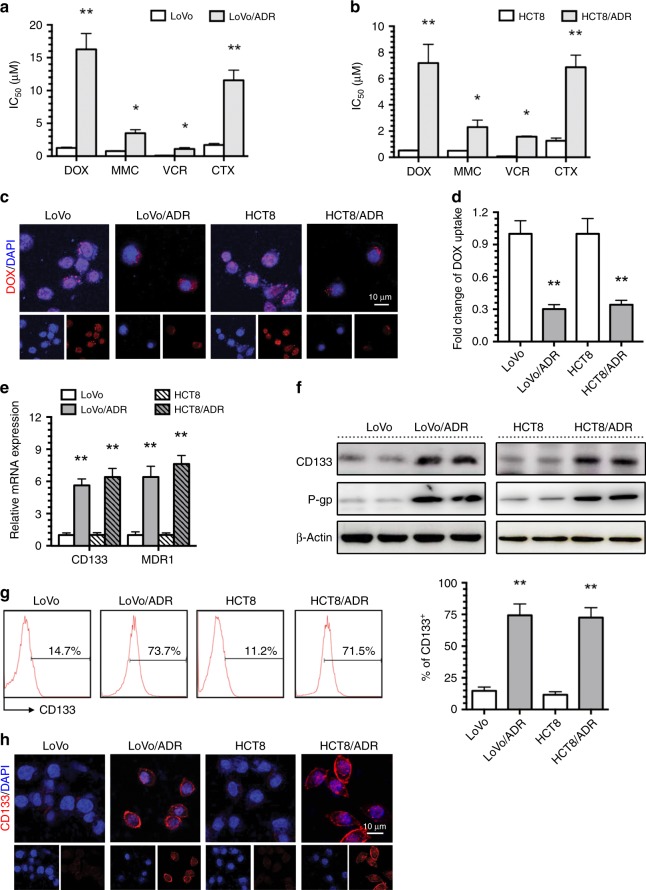
CD133 overexpressing in ADR-resistant CRC cells. **a**, **b** The IC50 values of DOX, MMC, VCR and CTX in LoVo and HCT8 CRC cells and LoVo/ADR and HCT8/ADR ADR-resistant cells were determined with a CCK-8 assay. **c** Intracellular distribution of DOX (red) in LoVo, HCT8, LoVo/ADR and HCT8/ADR cells 6 h after a 1 h incubation with 1 μg/ml DOX. **d** Quantitative DOX profiles over 6 h. **e**, **f** CD133 and MDR1/P-gp expression in LoVo, HCT8, LoVo/ADR and HCT8/ADR cells was determined by qPCR and western blotting. **g**, **h** CD133 expression in LoVo, HCT8, LoVo/ADR and HCT8/ADR cells was determined by flow cytometry and immunofluorescence (CD133: red; 4,6-diamidino-2-phenylindole, DAPI: blue). *p < 0.05, **p < 0.01. Each bar represents the mean ± SD of three independent experiments.

The Adriamycin (ADR)-resistant human CRC cells (LoVo/ADR and HCT8/ADR cells) that we used in previous studies displayed a significant MDR phenotype. ADR-resistant cell lines displayed strong cross-tolerance to mitomycin C (MMC), vincristine (VCR) and cyclophosphamide (CTX) (Fig. 1a, b) and significantly reduced accumulation of doxorubicin (DOX) (Fig. 1c, d). To investigate the relationship between MDR and CSC properties in CRC, we detected the CSC surface marker CD133 in ADR-resistant cells and their parental cells. CD133 and MDR1/P-gp expression increased in LoVo/ADR and HCT8/ADR cells compared to LoVo and HCT8 cells (Fig. 1e, f). Flow cytometry and immunofluorescence analyses showed that the expression of LoVo/ADR and HCT8/ADR cell surface markers increased with the emergence of drug-resistance phenotypes (Fig. 1g, h). This increase in CD133 expression indicates that the MDR phenotype may be related to CSC characteristics.

### CD133 is positively correlated with MDR1/P-gp in CRC

**Fig. 2:**
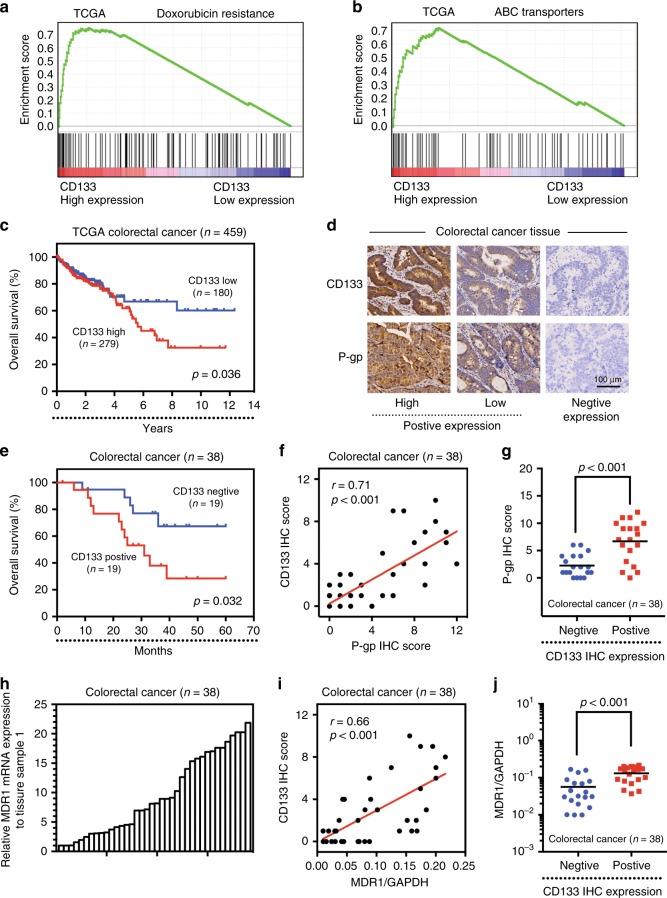
CD133 is positively correlated with MDR1/P-gp in CRC. **a**, **b** High expression of CD133 correlated with the DOX resistance signalling pathway and the ABC transporter-signalling pathway, as revealed using the TCGA datasets (*n* = 459). NES, normalised enrichment score. **c** Kaplan-Meier analysis of overall survival in patients with variable CD133 expression according to the data from the TCGA database (*p* = 0.036). **d** Expression of CD133 and P-gp protein was evaluated by IHC in selected CRC tissues (*n* = 38). **e** Kaplan-Meier analysis of overall survival in patients with variable CD133 expression according to data from selected CRC specimens (*p* = 0.032). **f**, **g** Expression levels of CD133 and P-gp are positively correlated among all the tissue samples. **h** Comparison of MDR1 mRNA expression levels in selected CRC specimens (*n* = 38). **i**, **j** Expression levels of CD133 and MDR1 mRNA are positively correlated among all the tissue samples (*n* = 38).

To explore the role of CD133 in the development of CRC, gene expression data from 459 cases were analysed from The Cancer Genome Atlas (TCGA) database. Kaplan-Meier survival curve analysis showed that the total survival time of patients with low CD133 expression was significantly longer than that of patients with high CD133 expression (Fig. 2c). In addition, gene set enrichment analysis (GSEA) of CRC tumours with high and low expression of CD133 from the TCGA database showed that CD133 expression was closely related to the DOX resistance signal transduction pathway and the ABC transporter signal transduction pathway (Fig. 2a, b). These results suggested that CD133 might be positively correlated with MDR1/P-gp, which is the core gene in both the DOX resistance signalling pathway and the ABC transporter-signalling pathway.

To confirm these results, immunohistochemistry (IHC) and qPCR were performed to assess CD133 and MDR1/P-gp expression in 38 CRC specimens (Fig. 2d, h). Kaplan-Meier survival analysis showed that the total survival time of CD133-positive patients was significantly shorter than that of CD133-negative patients (Fig. 2e). In this figure, each point in the scatter graph represents an individual sample, with the relative CD133 IHC score indicated on the *y*-axis and the MDR1/P-gp IHC score or the mRNA expression level indicated on the *x*-axis (Fig. 2f, i). The correlation coefficient showed that CD133 was positively correlated with P-gp protein expression (*r* = −0.71, *p* < 0.001) (Fig. 2f) and MDR1 mRNA expression (*r* = −0.66, *p* < 0.001) (Fig. 2i) in the 38 cases of CRC. The data were statistically analysed using the Spearman rank test. The results were consistent with the results presented above, and we further found that high expression of MDR1/P-gp was consistently accompanied by positive expression of CD133 (Mann-Whitney test, *p* < 0.001) (Fig. 2g, j).

### CD133 regulates MDR via the AKT/NF-κB/MDR1 signalling pathway in CRC

**Fig. 3:**
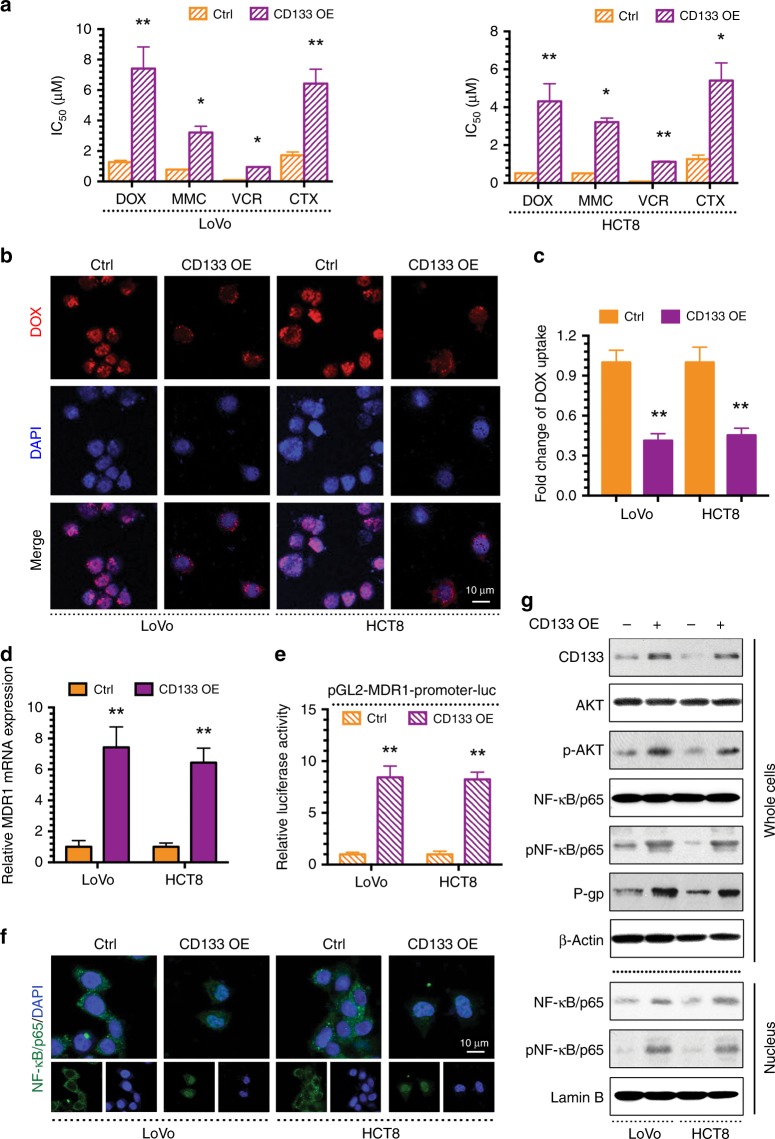
CD133 promotes MDR via the AKT/NF-κB/MDR1 signalling in CRC. **a** The IC50 values of DOX, MMC, VCR and CTX in LoVo, HCT8 and their CD133 overexpressing (CD133 OE) cells were determined with a CCK-8 assay. **b** Intracellular distribution of DOX (red) at the 6th h after 1 h incubation with 1 μg/ml DOX. **c** Quantitative DOX profiles over 6 h. **d** qPCR showing MDR1 mRNA expression. **e** Luciferase reporter assay showing MDR1 gene promoter activity. **f** Immunofluorescence showing the localisation of NF-κB/p65. **g** Western blots showing protein profiles of CD133, total AKT, p-AKT, total NF-κB/p65 (whole cells or nucleus), p-NF-κB/p65 (whole cells or nucleus) and P-gp. *p < 0.05, **p < 0.01. Each bar represents the mean ± SD of three independent experiments.

The abovementioned studies demonstrated that there is a positive correlation between the expression of CD133 and MDR1/P-gp in CRC patients. To confirm whether CD133 induces MDR1/P-gp-associated MDR in CRC, we transfected a CD133 overexpression (OE) plasmid into LoVo and HCT8 cells. Compared to their respective control cells, CD133 OE CRC cells displayed significant resistance to chemotherapeutic agents (DOX, MMC, VCR and CTX) (Fig. 3a) and markedly inhibited DOX uptake (Fig. 3b, c). Notably, MDR1 mRNA levels and MDR1 promoter activity were clearly increased in CD133-overexpressing cells (Fig. 3d, e). AKT signal transduction is related to MDR1 expression. Specifically, AKT activates NF-κB and further induces MDR1 expression by binding to its promoter. Inhibition of PI3K/AKT/NF-κB signal transduction can reduce the expression of MDR1. In addition, CD133 activates the AKT pathway in CD133-positive glioma stem cells. Therefore, we hypothesised that CD133 regulates MDR1 through the AKT/NF-κB axis, and immunofluorescence showed that NF-κB localised to the nucleus after CD133 OE plasmid transfection (Fig. 3f). The western blot results showed that CD133, p-AKT, p-NF-κB/p65 and P-gp levels were all increased in whole cells after CD133 OE plasmid transfection, and total NF-κB/p65 and p-NF-κB/p65 levels were increased in the nucleus (Fig. 3g). The above studies demonstrated that CD133 could regulate MDR by increasing MDR1/P-gp expression via induction of AKT phosphorylation, NF-κB/p65 nuclear translocation and MDR1 translation in CRC cells.

### Inhibiting CD133 reverses MDR via the AKT/NF-κB/MDR1 signalling pathway in CRC

**Fig. 4:**
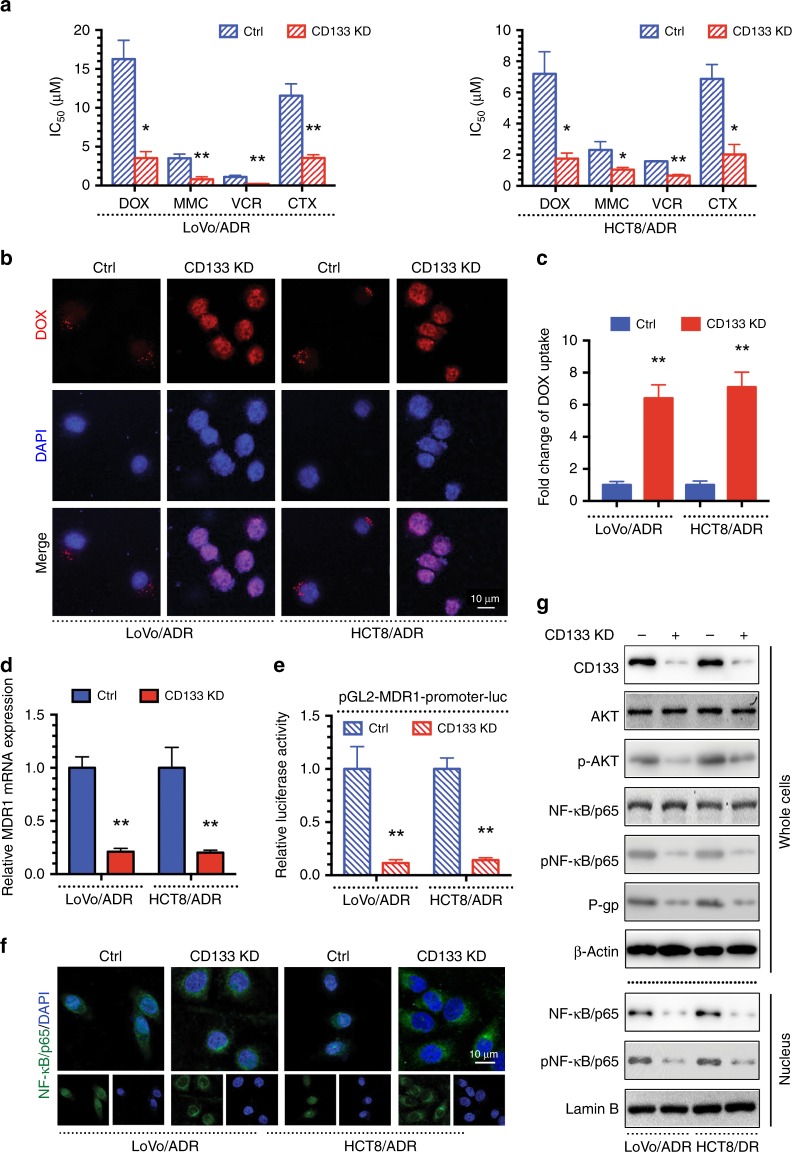
Inhibiting CD133 reverses MDR via the AKT/NF-κB/MDR1 signalling in CRC. **a** The IC50 values of DOX, MMC, VCR and CTX in LoVo/ADR, HCT8/ADR and their CD133-knockdown (CD133 KD) cells were determined with a CCK-8 assay. **b** Intracellular distribution of DOX (red) at the 6th h after 1 h incubation with 1 μg/ml DOX. **c** Quantitative DOX profiles over 6 h. **d** qPCR showing MDR1 mRNA expression. **e** Luciferase reporter assay showing MDR1 gene promoter activity. **f** Immunofluorescence showing the localisation of NF-κB/p65. **g** Western blots showing protein profiles of CD133, total AKT, p-AKT, total NF-κB/p65 (whole cells or nucleus), p-NF-κB/p65 (whole cells or nucleus) and P-gp. *p < 0.05, **p < 0.01. Each bar represents the mean ± SD of three independent experiments.

As shown here, CD133 regulates MDR via the AKT/NF-κB/MDR1 signalling pathway in CRC. Given this relationship, downregulation of CD133 should decrease MDR1 expression and increase tumour cell chemosensitivity. To investigate this hypothesis, drug-resistant LoVo/ADR and HCT8/ADR cells cultured in the presence of the drug they had acquired resistance to were treated with a CD133-knockdown (KD) plasmid. Downregulation of CD133 significantly sensitised MDR CRC cells to chemotherapeutic agents (DOX, MMC, VCR and CTX) (Fig. 4a) and markedly increased DOX uptake (Fig. 4b, c). Furthermore, inhibiting CD133 decreased MDR1 mRNA levels and promoter activity (Fig. 4d, e) and localised NF-κB in the cytoplasm of LoVo/ADR and HCT8/ADR cells (Fig. 4f). The western blot results showed that the MDR1-encoded protein P-gp, p-AKT, p-NF-κB/p65 and CD133 were reduced in MDR CRC cells treated with the CD133 KD plasmid, while total NF-κB/p65 and p-NF-κB/p65 levels were decreased in the nucleus (Fig. 4g). Overall, our results indicate that inhibiting CD133 expression reverses MDR by downregulating MDR1/P-gp via the AKT/NF-κB/MDR1 signalling pathway in MDR CRC cells.

### The AKT/NF-κB signalling pathway is the main mechanism by which CD133 regulates MDR1/P-gp expression in CRC

**Fig. 5:**
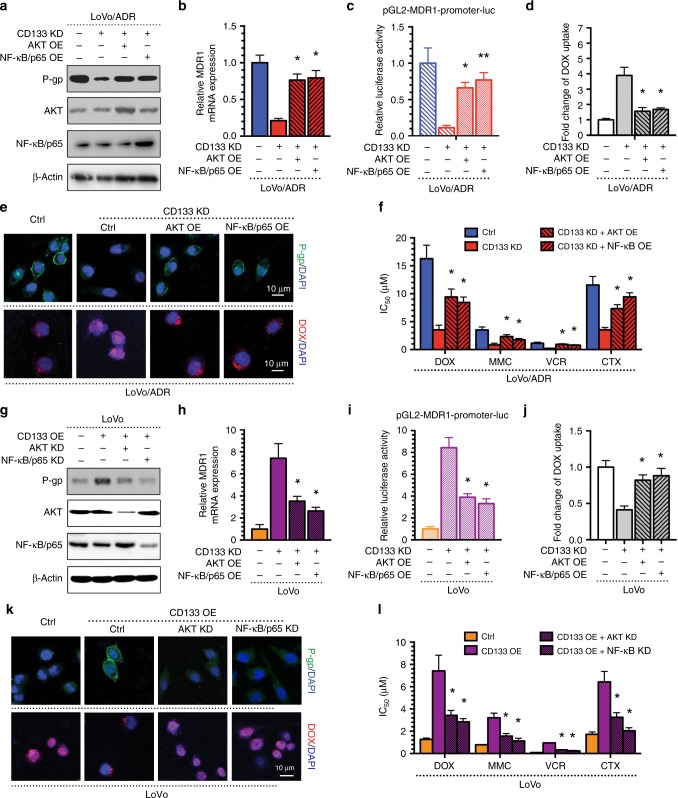
The AKT-NF-κB signalling pathway is the crucial mechanism of CD133 regulating MDR1/P-gp expression in CRC. The AKT OE plasmid or the NF-κB/p65 OE plasmid could reverse the effects of CD133 KD plasmid in LoVo/ADR cells: **a** reversal of reduced P-gp expression in western blots; **b** reversal of reduced MDR1 mRNA expression by qPCR; **c** reversal of reduced MDR1 gene promoter activity by a luciferase reporter assay. **d**, **e** reversal of enhanced intracellular DOX concentrations; **e** reversal of reduced P-gp expression by immunofluorescence; **f** reversal of enhanced sensitivity in the CCK-8 assay; The AKT KD plasmid or the NF-κB/p65 KD plasmid could reverse the effects of CD133 OE plasmid in LoVo cells: **a** reversal of increased P-gp expression in western blots; **b** reversal of increased MDR1 mRNA expression by qPCR; **c** reversal of increased MDR1 gene promoter activity by a luciferase reporter assay. **d**, **e** reversal of reduced intracellular DOX concentrations; **e** reversal of increased P-gp expression by immunofluorescence; **f** reversal of reduced sensitivity in the CCK-8 assay; *p < 0.05, **p < 0.01. Each bar represents the mean ± SD of three independent experiments.

To investigate whether the AKT/NF-κB signalling pathway is the main mechanism by which CD133 regulates MDR1/P-gp expression in CRC, we performed a rescue experiment in the MDR CRC cells LoVo/ADR. The western blot and immunofluorescence results showed that AKT or NF-κB/p65 could restore the decreased MDR1/P-gp expression induced by the CD133 KD plasmid (Fig. 5a, e). The same factors restored the expression of MDR1 mRNA and MDR1 gene promoter activity, as indicated by qPCR and a luciferase reporter assay, respectively (Fig. 5b, c). Moreover, AKT or NF-κB/p65 overexpression significantly decreased DOX uptake (Fig. 5d, e). Finally, the CCK-8 assay results showed that the AKT OE plasmid or the NF-κB/p65 OE plasmid could reverse the enhanced sensitivity of LoVo/ADR cells after CD133 KD plasmid transfection (Fig. 5f). Moreover, downregulation of AKT or NF-κB/p65 restored the increased MDR1/P-gp expression induced by the CD133 OE plasmid as demonstrated by western blot and immunofluorescence (Fig. 5g, k) and suppressed MDR1 mRNA levels and MDR1 gene promoter activity, which were enhanced by CD133 (Fig. 5h, i). Furthermore, AKT or NF-κB/p65 KD significantly increased DOX uptake (Fig. 5j, k). Finally, the IC50 of CRC cells to chemotherapeutic agents significantly decreased following AKT or NF-κB/p65 KD transfection (Fig. 5l). Taken together, these results suggest that CD133 regulates MDR in CRC cells through the expression of MDR1/P-gp, and the AKT-NF-κB signalling pathway plays an important role in this process.

### Inhibiting CD133 increases the antitumour effects of DOX in vivo

We observed that inhibiting CD133 reverses MDR by MDR1/P-gp in CRC cells in vitro. Next, we used in vivo experiments to verify this finding. We established a xenograft model of colon tumours by subcutaneous inoculation of isogenic LoVo/ADR cells expressing either control (ctrl) vector or CD133 KD plasmid into nude mice (each group, *n* = 5). After 2 weeks, the mice were divided into two groups. One group was treated with DOX every 5 days per week administered by i.p. injection and the other with the solvent control. After 4 weeks of treatment, tumour growth and therapeutic sensitivity were monitored until the mice were euthanised.

**Fig. 6:**
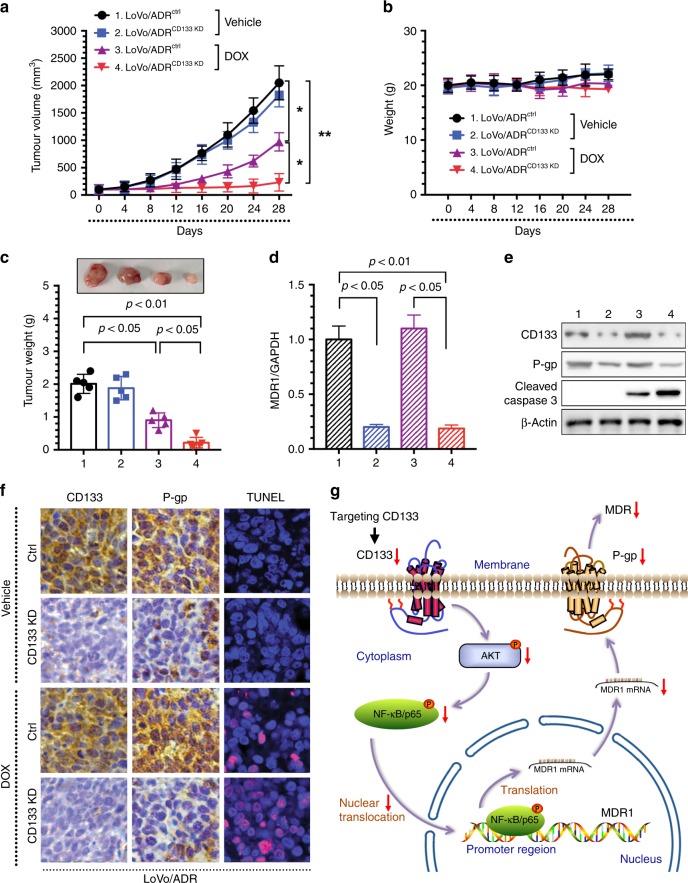
Inhibiting CD133 increases the antitumour effects of DOX in vivo. Inhibiting CD133 increased the effectiveness of DOX in the inhibition of tumour growth in vivo. **a** Xenograft tumour growth curves, **b** mouse weights and **c** pictures of tumours and tumour weights (Group 1: LoVo/ADR^ctrl^ + normal saline, Group 2: LoVo/ADR^CD133 KD^ + normal saline, Group 3: LoVo/ADR^ctrl^ + DOX, Group 4: LoVo/ADR^CD133 KD^ + DOX). **d** Inhibiting CD133 decreased MDR1 mRNA expression in vivo. **e** Inhibiting CD133 decreased P-gp expression and increased Cleaved-caspase 3 with DOX in vivo. **f** Inhibiting CD133 significantly increased apoptosis in response to DOX, as indicated by TUNEL, and reduced CD133 and P-gp expression levels in vivo. The images are representative of multiple fields of tumour sections from each group. The percentage of cells with positive TUNEL, CD133 and P-gp staining was determined as described in the Materials and Methods. *p < 0.05, **p < 0.01. Each bar represents the mean ± SD of three independent experiments. **g** Schematic diagram showing that targeting CD133 reverses drug-resistance via the AKT/NF-κB/MDR1 pathway in colorectal cancer.

After measuring the tumour volume, we plotted a growth curve of the transplanted tumours. The results showed that inhibiting CD133 significantly increased the sensitivity of LoVo/ADR cells to DOX (Fig. 6a), and the tumour size and tumour weight showed similar results, as expected (Fig. 6c). As shown in Fig. 6b, none of the test subjects lost body weight or died in any of the four groups at the doses tested, suggesting minimal toxicities. Moreover, the qPCR results showed that MDR1 mRNA expression was inhibited by the CD133 KD plasmid, as observed in vitro (Fig. 6d). To assess whether inhibiting CD133 sensitised the tumours to DOX and induced tumour growth regression via MDR1/P-gp in vivo, representative samples from harvested tumour tissues were analysed by WB and IHC for CD133 or P-gp expression, as described previously (Fig. 6e, f). Consistent with our in vitro observations, a discernible decline in P-gp was observed in tumour tissues of the CD133 KD group, and downregulation of CD133 increased the apoptotic effect of DOX (Fig. 6e, f). These data demonstrated that inhibiting CD133 could enhance the antitumour effect of chemotherapy agents in vivo.

## Discussion

MDR has become the major reason for chemotherapy failure in patients with CRC. Drug transporters of the ABC family are overexpressed in tumours, promoting resistance to chemotherapy drugs.^26^ MDR1/P-gp is a member of the ABC membrane transporter family that exhibits energy-dependent drug pump activity and can transfer drugs out of cells.^27^ Studies have shown that inhibition of MDR1/P-gp reverses drug resistance in CRC.^28^ However, direct treatment strategies for MDR1/P-gp have not proven successful in clinical trials. Therefore, in addition to MDR1/P-gp overexpression, other mechanisms undoubtedly contribute to MDR acquisition.

Recent studies have shown that CSCs are another important factor in the failure of chemotherapy.^5,6,29–31^ Compared to normal stem cells, CSCs have the ability to self-renew and differentiate and are characterised by the expression of specific stem cell markers.^5,6^ CD133 was characterised as a cell surface marker for CRC.^32,33^ Grillet et al. have found that circulating tumour cells from patients with colorectal cancer have cancer stem cell hallmarks in ex vivo culture.^16^ To investigate the relationship between MDR and CSC properties in CRC, we detected the CSC surface marker CD133 in ADR-resistant cells and their parental cells. The expression of CD133 and MDR1/P-gp was increased in ADR-resistant cells. The TCGA database analysis showed that CD133 expression was closely related to the DOX resistance signal transduction pathway and the ABC transporter signal transduction pathway. To better understand the potential clinicopathological implications of the relationship between CD133 and MDR1, we investigated the expression levels of CD133 and MDR1 in human CRC specimens. We found that CD133 was positively correlated with MDR1/P-gp, and that high expression of MDR1/P-gp was consistently accompanied by positive expression of CD133. To confirm whether CD133 induces MDR1/P-gp-associated CRC MDR, we constructed CD133-overexpressing (OE) and CD133-knockdown CRC cells and demonstrated that CD133 could regulate MDR by increasing MDR1/P-gp expression in vivo and in vitro.

The PI3K/AKT signalling pathway is closely related to MDR and blocking the PI3K/AKT pathway can lead to the downregulation of MDR1/P-gp protein expression, thus reversing MDR.^26^ When the cells are stimulated by external signals, the AKT protein is phosphorylated, leading to the phosphorylation of downstream IκB-α and its dissociation from NF-κB. After NF-κB nuclear translocation and binding to its recognition site, the promoter of the MDR1 gene is activated, and gene expression is induced.^17,18,34,35^ Tomita et al. found that CD133 regulates the level of MDR1/P-gp in glioblastoma through the PI3K/AKT-NF/κB signalling pathway, thereby promoting MDR.^36^ Li et al. reported that PKC DNA could regulate the expression of MDR1/P-gp in CD133-positive osteosarcoma cells through the PI3K/AKT/NF-κB pathway.^37^ These studies suggest that CD133 is able to regulate MDR1/P-gp through the PI3K/AKT pathway. However, there is no research on the molecular mechanism of the PI3K/AKT signalling pathway with regards to MDR and CD133 + CRC cell properties. We discovered that up- or downregulation of CD133 could regulate MDR via AKT/NF-κB/MDR1 signalling in CRC. To investigate whether the AKT/NF-κB signalling pathway is the main mechanism by which CD133 regulates MDR1/P-gp expression, a rescue experiment was performed in MDR CRC cells. Our results showed that AKT or NF-κB/p65 could restore the decreased MDR1/P-gp expression induced by CD133 KD, and downregulation of AKT or NF-κB/p65 restored the increased MDR1/P-gp expression induced by CD133 OE in CRC ADR cells. Taken together, these results suggest that CD133 regulates MDR through the expression of MDR1/P-gp, and the AKT-NF-κB signalling pathway is the main mechanism by which CD133 regulates MDR1/P-gp expression in CRC.

The targeted inhibition of these stem cell biomarkers is one of the promising approaches to eliminate cancer stemness.^38^ Ning et al. found that anti-CD133 antibody conjugated SN-38 nanoparticles could abolish the CSC population in CRC.^39^ Zhao et al. found that anti-CD133 monoclonal antibody (MS133) could successfully prevent tumour growth in CRC.^40^ Potentially, the most promising therapeutic implication of our findings is that inhibition of CD133 significantly increased the sensitivity of LoVo/ADR cells to DOX, and the tumour size and tumour weight showed similar results. It will be interesting to determine whether anti-CSC therapy combined with current antitumour drugs could be more effective against tumours.

In conclusion, we found that CD133 increased with the emergence of drug-resistance phenotypes, and the high expression of MDR1/P-gp was consistently accompanied by positive expression of CD133. Up- or downregulation of CD133 could regulate MDR via AKT/NF-κB/MDR1 signalling in CRC in vitro and in vivo. Importantly, analysis of patient samples showed that the expression of CD133 positively correlates with MDR1 in CRC. Furthermore, a rescue experiment performed in MDR CRC cells showed that the AKT/NF-κB signalling pathway is the main mechanism by which CD133 regulates MDR1/P-gp expression in CRC. Taken together, our results suggest that targeting CD133 reverses drug resistance via the AKT/NF-κB/MDR1 pathway and that this pathway might serve as a potential therapeutic target to reverse MDR in CRC.

## Data Availability

The datasets used and/or analysed during the current study are available from the corresponding authors on reasonable request.
